# Immunosenescence and Autoimmunity: Exploiting the T-Cell Receptor Repertoire to Investigate the Impact of Aging on Multiple Sclerosis

**DOI:** 10.3389/fimmu.2021.799380

**Published:** 2021-12-01

**Authors:** Roberta Amoriello, Alice Mariottini, Clara Ballerini

**Affiliations:** ^1^ Dipartimento di Medicina Sperimentale e Clinica (DMSC), Laboratory of Neuroimmunology, University of Florence, Florence, Italy; ^2^ Dipartimento di Neuroscienze, Psicologia, Area del Farmaco e Salute del Bambino (NEUROFARBA), University of Florence, Florence, Italy

**Keywords:** multiple sclerosis, T cell receptor (TCR), disease modifying therapies (DMTs), aging, autoimmune diseases

## Abstract

T-cell receptor (TCR) repertoire diversity is a determining factor for the immune system capability in fighting infections and preventing autoimmunity. During life, the TCR repertoire diversity progressively declines as a physiological aging progress. The investigation of TCR repertoire dynamics over life represents a powerful tool unraveling the impact of immunosenescence in health and disease. Multiple Sclerosis (MS) is a demyelinating, inflammatory, T-cell mediated autoimmune disease of the Central Nervous System in which age is crucial: it is the most widespread neurological disease among young adults and, furthermore, patients age may impact on MS progression and treatments outcome. Crossing knowledge on the TCR repertoire dynamics over MS patients’ life is fundamental to investigate disease mechanisms, and the advent of high- throughput sequencing (HTS) has significantly increased our knowledge on the topic. Here we report an overview of current literature about the impact of immunosenescence and age-related TCR dynamics variation in autoimmunity, including MS.

## Introduction

Immunosenescence is a natural consequence of the biological process of aging. The immune system progressively declines throughout life: the involution of thymic activity begins with puberty and, as age advances, the regenerative potential of immune cells decreases, skewing the T- and B-cell compartment (1, 2). Such changes reduce the immune system reactivity, making the individual more prone to infections and developing cancer.

T lymphocytes are profoundly impacted by immune aging. Over time, the T-cell compartment gradually switches towards a homeostatic maintenance of the existing cells rather than generating new ones, as reflected by the reduction of naïve cells (3, 4). In these circumstances memory T cells become prevalent, showing changes in either immunophenotype (5, 6) and gene expression (7, 8). The composition of the memory T-cell compartment in advanced age is closely linked to the individual immunological history, e.g. the infections acquired during childhood and adolescence. Viruses that infect the organism and then become lifelong latent have a major role in shaping the T-cell response and building-up the memory T-cell repertoire (9, 10). Cytomegalovirus (CMV) and Epstein-Barr virus (EBV) are two examples of pathogens that infect a massive part of the human population (global prevalence is up to 80% and 95% for CMV and EBV, respectively) (11, 12), leaving a signature within the immune system that is common to most of individuals and that, in some cases, alters the immune system composition as age progresses.

The application of high-throughput sequencing (HTS) demonstrated that age-dependent T-cell compartment depletion reflects the impairment of the T-cell receptor (TCR) repertoire diversity that appears reduced, leading to a narrowed antigen recognition potential breadth (4, 9, 13). Albeit this process naturally occurs in both health and disease, in the latter case it may exacerbate the pathological condition, weakening immune defense and capability of recovering (e.g. hampering to repair tissue damage and to heal from acute infections) and contributing to disease progression (14). This is particularly crucial in autoimmune disorders, in which the disruption of immune tolerance and immune aging are mutually related, as it has been observed in patients with Rheumatoid Arthritis (RA), who showed a disease-dependent premature and accelerated immunosenescence process (15).

Multiple Sclerosis (MS) is an inflammatory, systemic, heterogeneous disease in which autoreactive T cells migrate from the periphery to the central nervous system (CNS), leading to myelin disruption. MS is widely considered an autoimmune disease, however the antigen that triggers the abnormal immune response is still unknown. HTS is greatly contributing to our understanding of MS pathogenesis being a powerful tool to bridge molecular and clinical data, such as detecting longitudinal treatment-dependent signatures in the TCR repertoire of patients (16, 17). The relationship between aging and MS pathogenesis and progression is well known: immunosenescence and skewed T-cell compartment diversity in MS patients have been linked to a higher risk of developing a potentially fatal neurological disorder, the Progressive Multifocal Leukoencephalopathy (PML) (18); furthermore, patient’s age differentially impacts treatment outcome (19, 20). A recent study investigated the TCR repertoire dynamics in MS patients of different ages and enlightened interesting results showing that, despite the physiological decline of TCR repertoire diversity with age, this does not significantly differ between MS and healthy people (21).

In this Review, we summarize the current knowledge about immunosenescence and age-related TCR repertoire dynamics in autoimmune diseases, with emphasis on MS, and how HTS shaped the scientific perspective on this investigation.

## T-Cell Receptor Aging in Health: Overview of High-Throughput Sequencing Investigation

The imprecise V-J genes rearrangement generates the TCRβ chain, for a theoretical amount of about 10^15^ TCRβ sequences and a real estimate ranging between 10^6^-10^8^ (2, 22). The high variability of the TCRβ molecule mainly lies in the complementarity-determining region 3 (CDR3), the first responsible of the bond, with variable affinity, between the receptor and the cognate antigen. For this reason, the amino acid CDR3 sequence (CDR3-a.a.) is the target of election of HTS investigation.

**Figure 1:**
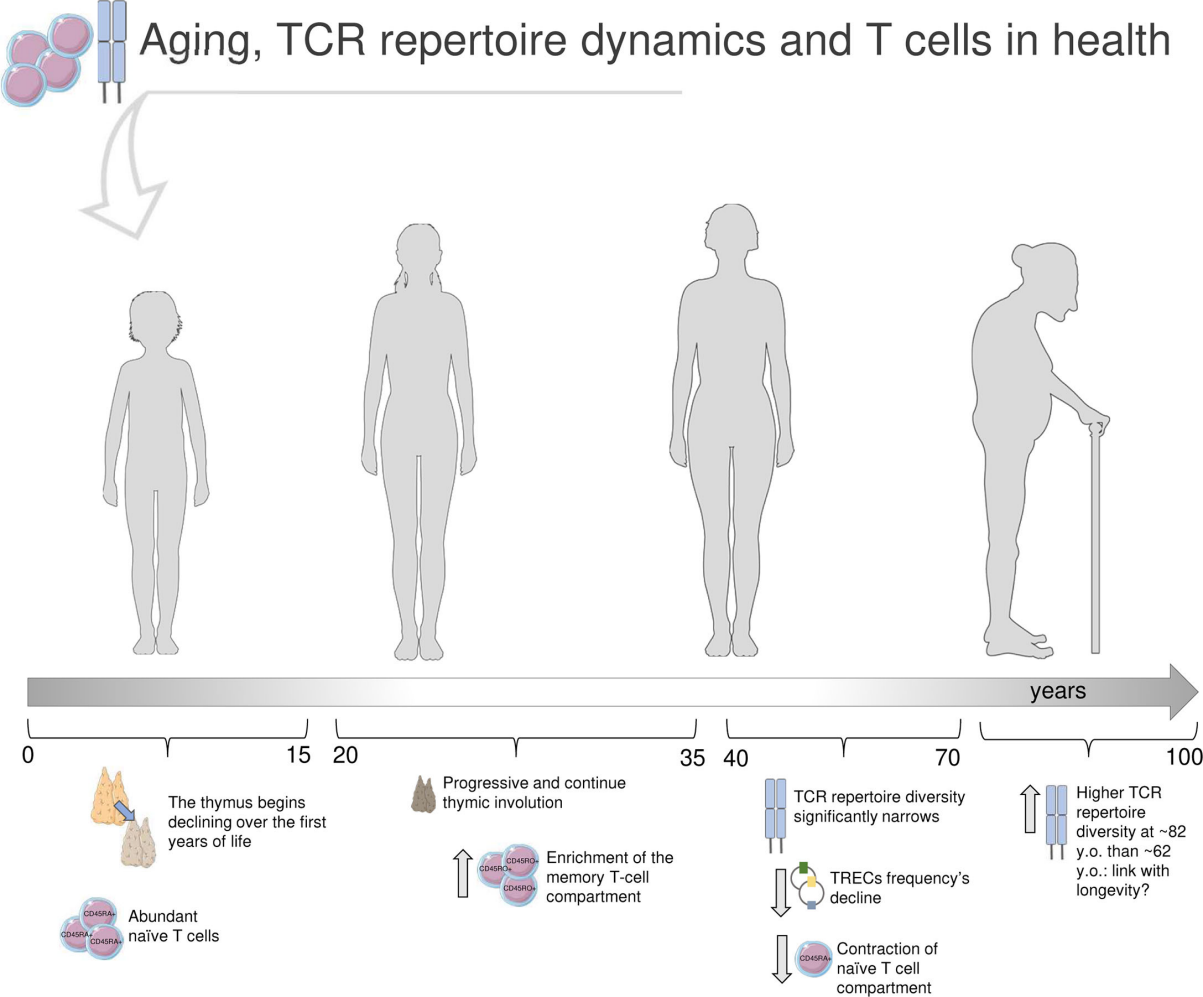
Immunosenescence and T-cell receptor repertoire in health. In the first years of life, the thymus is at its maximum size and activity, and the T-cell compartment is characterized by abundance of naïve (CD45RA+) CD4+ and CD8+ cells. Earlier in life, the thymus starts to shrink, and continues to decline over puberty and adulthood, alongside with changes in the T-cell compartment, now enriched in memory (CD45RO+) cells (23–27). The TCR repertoire diversity significantly narrows after the age of 40 years old (y.o.) (1, 2, 4). The strongly reduced thymic output reflects in a decline of T-cell receptor excision circles (TRECs) frequency, small molecules of circular DNA representative of T-cell maturation (27). Furthermore, the naïve T-cell compartment results significantly dwindled (23–27). Through the latest stages of life, some TCRβ clones, especially across memory repertoires, are definitely predominant; interestingly, Britanova et al. documented a higher TCR repertoire diversity in older people (average age of 82 y.o.) compared to 62 y.o. individuals, that may be suggestive of longevity (4). Parts of the image created using pictures adapted from Servier Medical Art (https://smart.servier.com/).

The process of thymic involution begins in the first years of life and progresses over puberty and adulthood (23, 24), determining a contraction of the T-cell compartment and a decrease of newly generated T cell: thus, naïve cells decline, memory T cells become prevalent, and TCR repertoire diversity dwindles (4, 25, 26). TCR repertoire richness starts to narrow noticeably from the age of about 40 (4); the naïve repertoire significantly declines in 70 years old (y.o.) adults, with 8-57 million different nucleotide sequences compared to the range of 60-120 million in young adults (20-35 y.o.) (2) (**Figure 1**: summary of aging’s impact on TCRβ repertoire in health).

Before the advent of HTS, aging’s impact on T-cell compartment was investigated in a large cohort of 156 healthy donors (HD) of different ages, by analyzing the frequency variation of T-cell receptor excision circles (TRECs), small molecules of circular DNA generated by thymic TCR genes rearrangement thus meaningful of T-cell maturation and used as markers of immunosenescence (27). The study reported that CD4+ cell compartment tends to be stable until the age of 70 years, then declines alongside with TCR diversity and TRECs frequency. Recent HTS studies (2, 4, 13) agree that the contraction of TCR repertoire richness, including a skewed peripheral Vβ family expression (8), is mainly observable in the naïve T-cell compartment. On the other side, effector memory CD8+ cells number increases with age, especially those cells that likely recognize latent viruses encountered over life by the majority of the population, such as CMV (2, 3, 28). Qi et al. showed that the age-dependent variation of effector memory T cells number in HD does not mirror the TCR repertoire richness dynamics, that does not significantly differ between young and elderlies, dissimilarly from what observed for naïve T cells (2). Accordingly, a previous study documented a linear decline of TCR repertoire diversity with age in peripheral blood naïve T cells of 39 HD (4). In fact, this decline was directly correlated with the percentage of naïve T cells, but not with the total count of circulating CD3+ cells, which did not show any age-dependent shrink. Britanova et al. observed that the oldest group of HD (average age of 82) was characterized by a broader TCR repertoire diversity compared to the group with average age of 62, suggesting that this molecular feature might be related with longevity. More recently, a longitudinal study (29) tracked the TCR repertoire dynamics over 20 years, in 6 HD of age ranging between 23-50 at the enrollment. The TCRβ gene was sequenced by HTS from peripheral blood CD4+ and CD8+ T-cell subsets for three times, about 10 years apart. According to other findings, TCR repertoire diversity dwindled more prominently in CD8+ cells, whereas CD4+ cells maintained a higher diversity constantly over time. Furthermore, authors found that the top of most frequent CDR3s-a.a. persisted over the whole period of observation (20 years), thus suggesting that a part of the TCR repertoire composition tends to remain stable over aging. Such findings may indicate that the TCR repertoire is strongly impacted by the encounter of specific antigens, that contribute to skew the repertoire towards a higher clonality and predominance of some Vβ clones rather than others (where a “clone” is a V-J-CDRβ3 a.a. sequence); this may be particularly marked in CD8+ cells, first actors in pathogen epitopes recognition, whereas peripheral CD4+ cells could be mostly sustained by homeostatic proliferation. Egorov et al. (13) performed HTS on HD peripheral blood TCRβ repertoire and, according to Qi et al. (2), observed a reduction of TCR diversity. In addition, authors detected an age-dependent reduction of the average CDR3 length and of the number of N nucleotides that are randomly added during TCRβ generation, and such decrease was correlated with the age-dependent involution of naïve T-cell proportion. Interestingly, authors suggest that a contracted CDR3 length may impact peptide antigen interaction, shrinking the antigen recognition breadth of the elderly immune repertoire.

Less is known about the TCRγδ repertoire dynamics over life. TCRγδ cells account for about 4% of human circulating T cells and are mainly enriched in intestine and spleen tissue as intraepithelial lymphocytes (IELs) (30). Different Vγ families alternate from birth until advanced age, varying in proportion over different stages of life (31, 32). Similarly to TCRαβ, TCRγδ repertoire encounters a contraction of diversity and an increased clonality with age, especially in some Vγδ families such as Vγ9+Vδ2+, and this is even prominent in naïve cells (33, 34). Since T γδ cells have important effector function in defense against pathogens and in bridging innate and adaptive immunity, their decline with age may contribute, along with T αβ cells, to a less active and less responsive immune system later in life.

## Senescent T-Cell Receptor Repertoire in Autoimmune Disorders: Progresses and Perspectives

**Figure 2:**
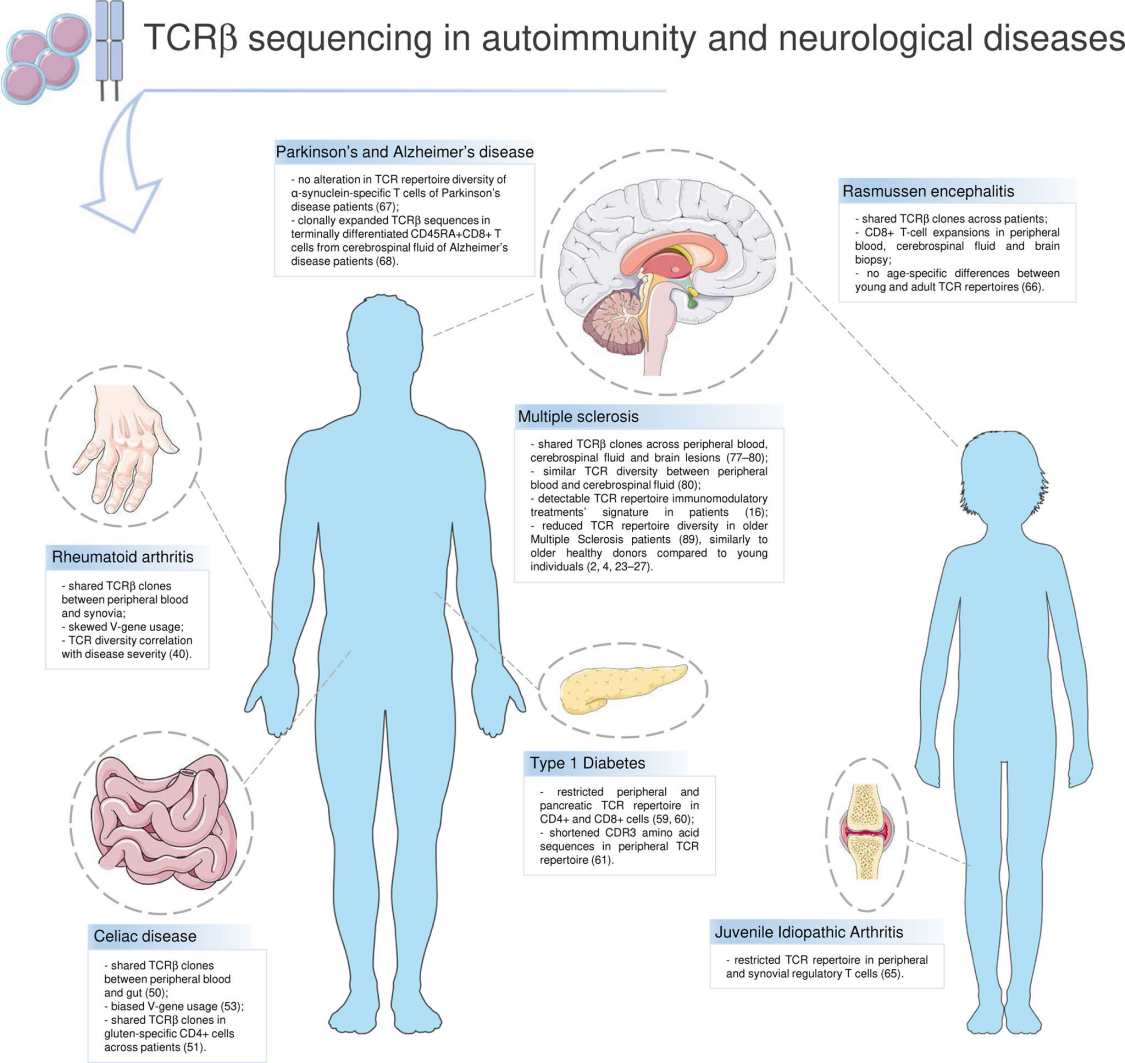
T-cell receptor repertoire in autoimmunity and neurological diseases. Highlights on current knowledge about the TCR repertoire and the T-cell compartment in autoimmune diseases (Rheumatoid arthritis, Celiac disease, Type 1 Diabetes, Multiple Sclerosis) and in other disorders in which T lymphocytes have a role (Rasmussen encephalitis, Juvenile Idiopathic Arthritis, Parkinson’s and Alzheimer’s disease). Parts of the image created using pictures from Servier Medical Art (https://smart.servier.com/).

T lymphocytes stage as main actors in a wide range of autoimmune diseases. Rheumatoid arthritis (RA), Celiac disease (CD), Diabetes Type 1 (T1D) and Multiple Sclerosis (MS) are main examples of autoimmune disorders in which T cells are crucial. In these diseases, the immune-mediated inflammation targets and damages different tissues, e.g. synovia in RA, small intestine in CD. Despite being a typical sign of autoimmunity, inflammation has been recently suggested to have a role in degenerative diseases as well, such as Alzheimer’s disease (AD) and Parkinson’s disease (PD) (35). HTS greatly contributed to characterizing the TCR repertoire in T-cell driven diseases and neurodegenerative ones (**Figure 2**).

*Rheumatoid arthritis.* Modifications in the T-cell compartment and TCR dynamics in RA are known since pre-HTS age (36). Synovial lesions infiltrates include mainly CD4+ T cells, that are characterized by an oligoclonal TCR repertoire and a narrowed TRBV families distribution (37–39). These findings have been recently implemented by HTS: Jiang et al. showed that RA patients share common features in their TCR repertoire, e.g. clonal expansion in the effector memory T-cell compartment and in T helper 17 (Th17) cells, and TCR diversity has been correlated with RA disease activity (40). Furthermore, authors detected abnormalities in the V-J gene usage that are shared between peripheral blood and synovial TCR repertoires, suggesting that autoreactive T cells might be activated and selected in the periphery and then infiltrate joints and *in situ* expand and contribute to inflammation. Immune aging has been discussed as a key factor in RA, despite contradictory findings being reported (41). T-cell compartment involution seems to be accelerated in RA patients, with increase of senescent CD28- T cells, characterized by declined proliferation and telomerase activity (42–44). In RA patients it was also documented a reduction of TRECs frequency; however, this was observed to be age-independent (45, 46), stressing the need for further investigation.

*Celiac disease.* CD is an autoimmune disease affecting the small intestine in which immune cells abnormally react against gluten proteins, causing inflammation and villous atrophy. Despite being a multifactorial disease, genetics plays a major role in CD susceptibility, in particular the human leukocyte antigen (HLA) DQ2 and DQ8 alleles, that are carried by up to 95% of patients (47, 48). CD4+ Th cells that react against gluten epitopes presented by HLA-DQ2.2, HLA-DQ2.5 and HLA-DQ8 are considered as main actors in CD pathogenesis and have been found in both peripheral blood and small intestine (49). HTS gave an extensive contribution gaining insights into T cells pathogenic role and TCR repertoire dynamics in CD patients. Comprehensive analysis on TCRVβ sequencing from CD patients documented the presence of sharing Vβ clones between peripheral blood and gut TCR repertoires (50) and the persistence of gluten-specific clones for decades in the TCR repertoire of patients upon gluten-free diet (GFD) (51). HTS on single-cell gut IELs TCRγδ showed that CD patients, either under GFD or not, have a biased TRDV pattern compared to healthy controls, a private CDR3δ repertoire, whereas CDR3γ clones are shared between CD and controls (52). A recent investigation leveraging a large TCRαβ dataset of data from 63 CD patients (53) identified a number of 325 public TCRαβ clones in gluten-specific CD4+ T cells across patient; furthermore, they observed a biased V-gene usage and conserved CDR3α:CDR3β motifs across CD repertoires. Taken together, these findings indicate that TCR repertoire shares common features across CD patients that may be linked to disease pathogenesis and progression; however, the association between TCR repertoire dynamics, T-cell senescence and aging in CD is still not deeply investigated. First, CD has long been considered almost exclusively a pediatric illness, whereas it can show an adult-onset; second, due to the wide variety of symptoms that can be subtle in some cases, CD remains an underdiagnosed disease (54). Therefore, future insights on TCR dynamics on CD patient cohorts of different ages are needed.

*Type 1 Diabetes.* T1D is an autoimmune disease of multifactorial and not yet totally understood etiology, in which autoreactive T cells attack pancreatic β-cells hampering insulin production and causing hyperglycemia, with a wide set of signs and symptoms of variable severity. Increased susceptibility to T1D is given by environmental (body mass index, nutrition habits, total weight, etc.) and genetic factors (55), the latter sustained by the comorbidity in T1D patients with other autoimmune diseases (e.g. CD) and the association with certain HLA class II haplotypes, such as HLA-DRB1, HLA-DQA1, and HLA-DQB1 (56). T1D onset is usually before the age of 40 years, with a peak between 15 and 20 years old, but it can occasionally be diagnosed in older patients (57). Notably, older (>40 y.o.) patients may show aggravated signs and symptoms compared to younger cohorts, and require special management, in particular for consequences of hyper- or hypoglycemia, cognitive impairment, and chronic pain (58). Tong et al. have been among the first investigating TCR repertoire in T1D patients by HTS (59), leveraging a TCRβ dataset from peripheral blood of nine T1D, four Type 2 Diabetes (T2D) and six non-diabetic controls. Authors described a skewed V-gene usage in T1D and a higher proportion of “highly-expanded clones” in T1D compared to other groups, considering “highly expanded” those T-cell clones with a frequency≥1% of total reads in a repertoire. In the same year, Seay et al. sequenced the TCR repertoire in different T-cell populations from various compartments, e.g. peripheral blood and pancreatic draining lymph nodes (pLN) (60). Differentially from what observed from Tong et al., Seay et al. did not find any skew of the V-gene usage in T1D compared to other groups; they described instead a restricted repertoire in CD4+ cells and a certain proportion (24%) of shared CD8+ clones among tissues. One year later, Gomez-Tourino et al. published a study in which the TCR repertoire was sequenced from peripheral T-cell subpopulations of 14 T1D patients and 14 HD (61); authors detected abnormalities in CDR3s-a.a. length of T1D, in particular highly frequent shortened CDR3s-a.a. shared across T1D repertoires. This abnormal sequence length, authors suggested, may depend on early events in thymic selection in T1D, and shortened CDR3s-a.a. may facilitate the erroneous recognition of self-antigens. In the abovementioned studies, however, the enrolled cohorts have a quite homogeneous average age, and TCR repertoire dynamics is not described from an age-related perspective.

## Neurodegenerative and T-Cell Mediated Diseases Affecting Young or Elderly

### Juvenile Idiopathic Arthritis and Rasmussen Encephalitis

It is worth mentioning a couple of comprehensive and recent TCR repertoire HTS studies that were performed in diseases usually affecting the pediatric population and in which T cells have an established role, despite these diseases are not frankly classifiable among autoimmune ones. This is the case of Juvenile Idiopathic Arthritis (JIA) and Rasmussen encephalitis (RE). The first is a rheumatologic disease with usual onset before the age of 16 years and of unknown etiology, in which pathogenesis T cells surely contribute, as demonstrated by the presence of activated T cells in synovial fluid and the effectiveness of treatments targeting T cells (62). RE is a progressive, chronic and inflammatory brain disease that mainly affects 6-7 y.o. children. RE pathogenesis is controversial and still under debate; however, it has been documented the presence of T cells, mainly CD8+, infiltrating the brain of patients (63, 64).

In JIA patients of age ranging between 4.9 and 15.1 years, the TCR repertoire of regulatory T cells (Treg) was restricted and expanded in both peripheral blood and synovial fluid (65). In RE patients, Schneider-Hohendorf et al. described CD8+ T-cell expansion in peripheral blood, cerebrospinal fluid (CSF) and brain biopsy, and Vβ clones and V-genes shared across patients (66). Authors analyzed TCR repertoire dynamics dividing patients between early (age range of 3-16 years) or adult (age range of 19-59 years) onset, but did not detect any age-specific difference between the two groups.

### Parkinson’s and Alzheimer’s Disease

PD and AD are two neurodegenerative disorders mainly affecting the older part of the population, with an average onset of 60 y.o. for PD and over 65 y.o. for AD, in which the potential pathogenic role of T cells has been recently discussed (35). The TCR repertoire was investigated in T cells reactive to self-antigen α-synuclein (α-syn), associated with the disease, from six PD patients, and compared with the repertoire of T cells reactive to pertussis, encountered over life by most of the population (67). Interestingly, the TCR repertoire diversity of PD was similar to pertussis-reactive T cells. In AD patients, the TCR repertoire was recently investigated by Gate et al. who published their data in 2020. By single-cell technology, they sequenced the TCR repertoire from effector memory terminally differentiated (Temra) CD45RA+CD8+ T cells of AD CSF and found clonally expanded clones that are probably responsive to EBV antigens (68).

It is thus clear that some of the mentioned diseases differ for incidence, severity and progression based on age ranges within the population, and that TCR repertoire investigation by HTS widened our understanding of underlying mechanisms; however, the link with age is not established or not discussed in most of TCR studies, and further insights are required.

## T-Cell Receptor Repertoire and Aging in Multiple Sclerosis

MS is currently the most widespread potentially disabling neurological disease among young adults, with an average age of onset of 35 y.o (69). MS can manifest through different patterns, of which the Relapsing-Remitting (RRMS) is the most common one (85% of all MS diagnosis) and characterized by a relatively more benign course compared to Primary-Progressive MS (PPMS) onset. Usually, younger patients show a RRMS course; furthermore, women are affected more often than men, with a ratio of approximately 3:1 (70). Despite the undetermined etiology, it is known that an interplay of environmental and genetic factors contributes to MS susceptibility, the first including infections acquired during childhood and adolescence (e.g. EBV and CMV), smoking, vitamin D deficiency, whereas HLA genes are well-known among genetic factors, such as DRB1*15:01 and DRB5*01:01 alleles in European population (71).

T lymphocytes have an established role in MS pathogenesis (72). It is believed that T cells activated in the periphery migrate through the blood-brain barrier (BBB) into the CNS, leading to demyelination. Since the antigen that triggers the autoreactive T-cell response is still unknown, the investigation of TCR repertoire has been a great point of interest years before the advent of HTS. The first TCR studies were conducted exploiting flow cytometry analysis on Vβ families or spectratyping technology, that allowed to investigate TRBV families clonal prevalence in terms of CDR3 length distribution, but not at sequence level. Pre-HTS studies detected a skewed TRBV families distribution in MS peripheral blood (73), clonally expanded CD8+ T-cell clones in MS brain lesions (74), CSF (75, 76) and blood (76). Later HTS studies allowed to track the presence of shared CDR3s-a.a., in MS across peripheral blood and CNS compartments (CSF and brain) (77–79). In particular, researchers found expanded Vβ clones, especially in the CD8+ cell compartment, being shared across blood, CSF and brain lesions of patients (78, 79). Accordingly, a recent comprehensive analysis pooling MS TCR sequencing data from published and unpublished studies (80) showed that blood, CSF and brain lesions share sequences, despite CSF repertoire is overall more private compared to the periphery; furthermore, CSF and blood are quite similar in terms of TCR repertoire diversity, that does not significantly differ between the two compartments.

Several studies suggested that MS pathogenesis may be linked to premature immunosenescence, and that aging could in turn impact disease progression, severity, and treatment outcome. This topic has been extensively reviewed by Dema et al. (81), who recapitulated recent findings about aging and MS: patients showed signs of premature immunosenescence such as shortened telomeres (82), thymic dysfunction (83), decreased TRECs frequency (84), and accumulation of CD4+CD28- T cells (85). In this frame, Dema et al. also discussed therapeutic strategies based on rejuvenating senolytics that showed promising results in mice (86, 87).

Recently, Hayashi et al. (21) characterized the TCR repertoire by HTS in peripheral blood of 39 MS and 19 HD by using the newly developed Grouping of Lymphocyte Interactions by Paratope Hotspots (GLIPH), a clustering method that allows to group data based on chosen parameters, e.g. TCRVβ chain distribution or HLA haplotype. To avoid any potential treatment-dependent bias on the TCR repertoire, authors excluded from the study patients undergoing treatments that may perturbate the TCR repertoire, such as fingolimod and natalizumab. These two treatments act specifically on T cells: the target of fingolimod is the sphingosine-1-phosphate receptor (S1PR), that is fundamental for T cells egress from lymph nodes and interacts with the C-C chemokine receptor type 7 (CCR7), involved in T cells homing to secondary lymphoid organs. Fingolimod induces the internalization of the S1PR, that binds CCR7+ T cells and causes their retention within lymph nodes. Natalizumab is a humanized monoclonal antibody targeting the integrin α4β1, or very late antigen-4 (VLA4), and blocking transmigration of T lymphocytes across the BBB. Both treatments are known to have immunomodulatory effects on T cells phenotype and TCR repertoire (ref. par. 5). In Hayashi et al., most (66.7%) of the enrolled patients were free from any therapy; the rest were under interferon-beta (20.5% of patients), prednisolone (10.5%) and azathioprine (2.6%). Authors analyzed data with regard to age, comparing MS with HD (age ranges of 40-63 and 43-59, respectively), and showed that older participants were characterized by lower TCRαβ and γδ diversity, with no significant differences between HD and MS. Interestingly, MS repertoires showed an overall age-independent broader TCR diversity compared to HD; such findings are in agreement with a previous study reporting a higher TCR diversity in MS in respect to HD and to a group of patients affected with a non-autoimmune neurological inflammatory disease, the Human T-cell leukemia virus type 1 associated myelopathy/tropical spastic paraparesis (HAM/TSP) (88).

## Disease-Modifying Therapies and T-Cell Receptor Repertoire in Multiple Sclerosis

Disease-modifying therapies (DMTs) for MS encompass immunomodulatory or immunosuppressive treatments reducing the auto-reactivity of the immune system and promoting an anti-inflammatory environment (89, 90). Main data collected so far on DMTs, TCR repertoire and MS are summarized in **Table 1**.

**Table 1:** Disease-modifying treatments, TCR repertoire and immunosenescence in Multiple Sclerosis.

Treatment	Route of administration	Mechanism of action	Effect on TCR repertoire	Link with immunosenescence
**AHSCT**	One-shot treatment consisting in (1) mobilisation of HSC from bone marrow (2); conditioning: leukapheresis and reinfusion of HSC after immunoablation by chemotherapy.	Immune system renewal and immune tolerance restoration.	- Broader TCR diversity (16, 91); - Effective reconstitution of CD4+ TCR repertoire (91); - Persistence of clonally predominant CD8+ T-cell clones (91); - Narrowed TCR diversity (91) and lower clonal persistence at 24 months from transplantation (16) correlated with treatment outcome and disease activity; - Public TCRβ clones shared across AHSCT patients after 24 months from treatment (16).	Increase of TRECs frequency and thymus reactivation after AHSCT (91, 92).
**Alemtuzumab**	Infusion in two cycles, one year apart.	Binding of CD52 with depletion of T and B cells and consequent repopulation.	Restricted TCR repertoire, especially in CD8 (93, 94).	- Reduction of TRECs frequency at 6 months of treatment, then return to basal levels; no TRECs-age correlation detected (95).
**Glatiramer acetate**	Subcutaneous injection daily or thrice a week.	Immunomodulant mimetic of myelin basic protein, attracting autoreactive T cells	No significant differences in TCR repertoire restriction compared to HD or pre-treatment (95).	Not documented.
**Fingolimod**	Oral, daily.	Causes S1PR internalization and retains T cells within lymph nodes.	Increasing TCR restriction over 12 months of treatment (spectratyping analysis (96);	Reduction of TRECs and KRECs frequency (95, 96); KRECs frequency, but not TRECs, was positively correlated with age (97).
**Natalizumab**	Infusion every four weeks.	Binding of VLA-4 integrin, blocking T-cell migration through the BBB.	- Oligoclonal TCR repertoire in CSF of treated patients (spectratyping analysis (98); possible connection with impaired CNS immuno surveillance and higher risk of PML; - Persistence of clones, especially memory CD8+, in peripheral blood of RRMS afer 24 months of treatment (16); - Broader TCR sequence similarity architecture in peripheral blood compared to one-shot treatment (AHSCT) after 24 months of natalizumab (16).	- Increase of TRECs frequency at 6 and 12 months of treatment, positively correlated with age (95); - Restricted TCR repertoire and decreased expression of CD49d, more pronounced in older patients (99).

Data on the impact of DMTs on TCR repertoire were first provided by studies on patients treated with autologous hematopoietic stem cell transplantation (AHSCT). AHSCT is a one-shot treatment (e.g. the administration of any DMTs is not required following the procedure, unless a disease reactivation is observed) consisting of four main steps. Briefly, hematopoietic stem cells (HSCs) are mobilized from the bone marrow by the administration of granulocyte-colony stimulating factor associated with cyclophosphamide (mobilization). The hematopoietic stem cells are then collected with leukapheresis and reinfused following the administration of high-dose chemotherapy (conditioning). Different drugs can be administered as a conditioning protocol, and conditioning regimens are classified in three grades of intensity (low, intermediate or high), according to the increasing grade of immunoablation induced (100). The immunoablation and the subsequent reconstitution promoted by the reinfusion of the HSCs induce a renewal of the immune system with a restoration of the immune tolerance. This latter phenomenon explains the long-term suppression of new inflammatory disease activity (relapses, and new T2 or gadolinium enhancing lesions) observed following transplant, in the absence of any maintenance therapy (101, 102). AHSCT has recently been endorsed as “standard of care” for the treatment of highly active RRMS failing DMTs by the European Committee for Blood and Marrow Transplantation (EBMT) guidelines (100), and its superior effectiveness compared to DMTs in RRMS patients was recently demonstrated by a randomized clinical trial (103). Younger age at treatment was independently associated with a reduced risk of disability progression following AHSCT in a retrospective multicenter cohort study (17), but this association is probably due to the predominance of different main drivers of disability progression according to age at treatment (i.e. disability accrual mostly inflammatory-driven in younger individuals than neurodegeneration driven in older individuals), rather than to a potential age-related variations in immune reconstitution following AHSCT.

The extensive study of variation of the TCR repertoire in patients treated with AHSCT provided valuable insights into the mechanism of action of the procedure, suggesting that the procedure was able to induce a re-booting of the immune system. Analysis of TRECs suggested that thymic reactivation could take place in adult individuals undergoing AHSCT, with the generation of new T cells following positive selection and maturation in reactivated thymus. Moreover, the reconstitution of an overall broader clonal diversity and an extensive renewal of clonal specificities compared with the pre-transplant assessment was first reported adopting CDR3 spectratyping (92). In a more recent study, HTS was applied to sequencing the TCRβ chains of 1 million sorted CD4+ and CD8+ T cells from each patient before transplant and 1 year after transplant (91). Impact of the procedure was different on CD4+ and CD8+ T cells: in CD4+ T cells, dominant TCR clones present before treatment were undetectable following the reconstitution, and patients largely developed a new repertoire. In contrast, dominant CD8+ clones were not effectively removed, and the reconstituted CD8+ T cell repertoire derived from clonal expansion of cells already detectable before treatment. Notably, patients who failed to respond to treatment showed less diversity in their TCR repertoire early during the reconstitution process, suggesting that this step is crucial for the successful outcome of the procedure. More recently, HTS was applied in paired blood and CSF samples from patients treated for active RRMS, comparing the reconstitution of T cell composition in both compartments before AHSCT and up to 4 years following transplantation (104). More than 90% of the pre-existing CSF repertoire was removed following AHSCT and replaced with Vβ clones predominantly generated from engrafted HSCs. Of the pre-existing clones in CSF, approximately 60% were also detected in blood before therapy, and concordant treatment effects were observed for Vβ clones in both compartments following AHSCT. Overall, these results indicate that replacement of the pre-existing TCR repertoire in active RRMS is a mechanism for AHSCT efficacy, suggesting that TCR analysis might be adequately performed in peripheral blood as a surrogate for CSF.

The impact of AHSCT on TCR repertoire was furtherly investigated and compared with natalizumab in a recent HTS study (16): 15 RRMS patients’ TCR repertoire dynamics was monitored longitudinally, before and after 24 months from AHSCT or under natalizumab, in peripheral naïve and memory T-cell subpopulations. The investigation detected treatment-specific signatures in RRMS TCR repertoire; in particular, authors found that AHSCT and natalizumab differentially impacted on TCR clonal expansion state, clonal persistence over time, and TCR repertoire architecture, and that such effects are traceable by comprehensive molecular approaches.

Accordingly with findings in MS (91, 104), successful AHSCT outcomes were correlated to an increased TCR repertoire diversity also in systemic sclerosis (SSc) (105, 106), a skin autoimmune disease, whereas SSc patients who experienced post-AHSCT relapse showed a reduced TCR repertoire diversity (107).

A few studies investigated the variation of TCR repertoire in MS patients treated with other DMTs such alemtuzumab, fingolimod, natalizumab and glatiramer acetate. Two studies reported data on thymic output and TCR repertoire in patients treated with alemtuzumab (93, 94, 108, 109). Alemtuzumab is a humanized monoclonal antibody targeting CD52, a surface marker primarily expressed on T and B lymphocytes, and inducing lymphocytic depletion followed by subsequent repopulation (109). Alemtuzumab is administered in two cycles, one year apart, and a re-treatment during year 3 might be administered in cases with persistent disease activity. In both the studies on alemtuzumab, TREC numbers were reduced following each course of treatment. The TCR repertoire was explored by CDR3 spectratyping and a more pronouncedly constricted TCR repertoire compared to baseline was detected following each cycle, and this was more pronounced in CD8+ cells (93). These data indicate that repopulation of T cells following the depletion induced by alemtuzumab is promoted by homeostatic proliferation of cells that have escaped depletion, rather than by newly generated cells from the thymus, suggesting that thymopoiesis is not significantly induced by alemtuzumab treatment.

Similarly, a narrowed TCR diversity in peripheral CD8+ cells of alemtuzumab-treated RRMS patients has been recently confirmed by a HTS study (94); these patients were characterized for the presence of highly active CD8+ cells in peripheral blood and infiltrating derma and developed vitiligo 14, 18 and 52 months after starting the treatment, therefore suggesting that the kinetics of B cells reconstitution and narrowing of the TCR repertoire might be involved in the development of secondary autoimmunity observed following alemtuzumab treatment (93, 94).

Fingolimod is a DMT that successfully reduces relapse rate and disease activity in relapsing MS (110). A longitudinal CDR3 spectratyping investigation detected the presence of TCR restrictions in peripheral blood of MS patients even before starting the treatment, which were then increased after 12 months of fingolimod (96). Furthermore, the study reported a significant reduction, over treatment, of TRECs and K-deleting recombination excision circles (KRECs) frequency in peripheral blood of patients.

A study adopting CDR3 spectratyping on patients treated with natalizumab (111) showed that during treatment patients exhibited a lower proportion of Vβ elements with TCR repertoire expansions in blood compared to non-natalizumab treated MS patients, but this phenomenon appeared to reverse in cases who developed PML (98). In the CSF, the TCR repertoire was more skewed or oligoclonal compared to corresponding blood samples, and these alterations in CSF were more prominent in patients treated with natalizumab compared to non-natalizumab-treated MS patients. The marked restriction of the TCR repertoire in the CSF of natalizumab-treated patients was suggested to critically weaken CNS immune surveillance exerted by patrolling memory T cells, potentially promoting the onset of PML in a proportion of John Cunningam virus (JCV) positive patients.

On the other hand, no significant differences by CDR3 spectratyping were detected in TCR repertoire of patients treated with glatiramer acetate compared to the pre-treatment, and the small variations reported in MS patients following start of treatment with glatiramer acetate were similar to those observed in healthy controls in the same study (112).

The potential additive effect of DMTs on premature immunosenescence and aging in patients with MS was evaluated in a few studies, exploring correlations with adverse events.

Paghera et al. quantified TRECs and KRECs in 122 MS patients aged from 17 to 60 years who had started therapy with interferon-beta, fingolimod, alemtuzumab, or natalizumab, measured in samples obtained before the therapy and at months 6 and 12 of treatment (95). TRECs and KRECs were used as surrogate markers of a senescent phenotype, as they are considered as indicators of thymic and bone marrow output (97). In therapy-naïve patients, the number of newly produced T and B cells was inversely correlated with age. The DMTs analyzed induced opposite changes in the production of new T and B cells, aligned with the known mechanism of action. The abovementioned correlation found at baseline was still detectable at month 12 of therapy with interferon-beta or natalizumab. On the other hand, both the correlations were lost in patients treated with alemtuzumab due to the reduction in TRECs and increase in KRECs, while in fingolimod-treated patients, only the correlation between TRECs and age disappeared. Overall, these data suggest that some DMTs might accelerate the immunosenescence of T cells, with potential increase of side effects mostly in elderly patients, aligned with the observation of higher risk of opportunistic infections during treatment in this latter population compared to young individuals (113).

Furthermore, the potential additional effect of DMTs on immunological changes induced by ageing has been suggested to play a role in the development of PML in MS patients receiving DMTs (18). The restriction of the TCR repertoire induced by natalizumab, further narrowing the TCR restriction induced by age, and associated with the impairment of T cells patrolling the CNS induced by the reduced transendothelial migration due to a decreased expression of CD49d (99), might in part explain the higher risk of PML and the worse outcomes reported in older patients compared to younger ones (114). Increased risk of PML in patients receiving fingolimod was associated with immune system changes induced by the treatment similar to those occurring during aging, as a predominance of Temra over naïve T cells (115, 116).

Collectively these data suggest that the risk of opportunistic infectious adverse events is increased in individuals with evidence of immunosenescence and that DMTs might exert an additional effect on the immune system of such individuals, considerably increasing this risk. Moreover, as DMTs might induce long-term effects on the immune system, persisting also following drug discontinuation, the actual immunological age of the individual might be persistently affected by previous treatments, thus making treatment switches challenging.

## Discussion

Autoimmune diseases are widely spread worldwide and affect people of all ages. Tracking TCR repertoire dynamics in humans is crucial to shed light on immunosenescence and its role in disease progression and treatments outcome; in this respect, HTS is greatly contributing.

To date, the majority of HTS studies on TCR repertoire in immunosenescence has been performed in healthy individuals. Recent findings highlighted that the healthy TCR repertoire is strongly impacted by antigens encountered over lifetime and consequently dwindles (3, 4, 10, 12), and such variations can be better appreciated in memory T cells after the age of 40 (4); furthermore, the shrinkage of TCR repertoire diversity correlates with the natural involution of the naïve T-cell compartment (4, 7, 13). TCR repertoire analysis in elderly also showed great potential unrevealing molecular markers of longevity, as Britanova et al. (4) found that healthy people older than 80 years are characterized by a broader TCR repertoire diversity compared to individuals of 65 y.o.

On the other hand, the current knowledge about immunosenescence and TCR repertoire dynamics in autoimmune and T-cell mediated diseases is still quite fragmented. In RA (15, 36–39), CD (49–53) and T1D (59–61) patients, different compartments (blood, synovia, gut, pancreatic draining lymph nodes) share TCRβ clones and show reduced TCR repertoire diversity. Furthermore, immunosenescence seems to be accelerated in RA patients (15, 42–44), and T1D severity is aggravated in older patients (58); however, most of these studies are not centered on an age-related perspective, stressing for further insights. It is worth mentioning that in Rasmussen encephalitis, a mainly pediatric disease of uncertain etiology but in which T cells have an established role, no correlation was found between TCR repertoire dynamics and patients’ age (66).

In MS, the relationship between the TCR repertoire and aging is complex and distinguishes patients undergoing treatments that do not perturbate the TCR repertoire (e.g. interferon-beta) and patients under DMTs. In the first case, the TCR repertoire diversity declines with age similarly to HD, suggesting that the age-dependent involution of the T-cell compartment may not be among hallmarks of MS (21). On the other hand, variations of the TCR repertoire are observed during physiological aging and might be induced by the administration of DMTs in people affected by MS. The generation of a different and wider TCR repertoire compared to that one detected prior to treatment has been widely demonstrated in MS patients treated with AHSCT, and this is thought to mediate the therapeutic effect of the procedure through the reconstitution of a newly tolerant immune system, promoted also by the reactivation of thymopoiesis (91, 92). Alemtuzumab induces further restriction of the TCR through a homeostatic proliferation-based immune repopulation (93, 94, 109). Divergent effects on the TCR and TRECs are induced by other DMTs (96, 98, 112). All these observations were reported in the general MS population; to our knowledge, no data are available so far on the impact of DMTs on TCR in aging, and the differential effectiveness of DMTs across classes of age is mainly attributable to the different mutual relationship between inflammation and neurodegeneration underlying the accrual of disability.

The effect of DMTs on the immune system of MS patients, promoting in some cases the development of changes similar to those induced by physiological immunosenescence, might increase the risk of potential side effects, mostly concerning opportunistic infections (18, 99, 114). The long-term effects on the immune system induced by DMTs and the potential additive effect on an immune system already showing features consistent with immunosenescence requires careful consideration of the individual characteristics to minimize potential detrimental effects of treatment.

In conclusion, the TCR repertoire investigation by HTS in healthy individuals has widened our understanding of how the adaptive immune system dynamics changes over lifetime. Furthermore, recent findings pointed out that immunosenescence impacts disease pathogenesis, including MS, and how patients respond to therapy. However, most of the studies do not focus on the relationship between aging and TCR dynamics, stressing for further insights. Deepening this investigation might be, in the future, an interesting tool for understanding disease mechanisms and customizing therapies to each individual patient.

## Author Contributions

RA conceived and wrote the manuscript. AM contributed in writing the manuscript. CB conceived and supervised the writing of the manuscript. All authors contributed to the article and approved the submitted version.

## Conflict of Interest

The authors declare that the research was conducted in the absence of any commercial or financial relationships that could be construed as a potential conflict of interest.

## Publisher’s Note

All claims expressed in this article are solely those of the authors and do not necessarily represent those of their affiliated organizations, or those of the publisher, the editors and the reviewers. Any product that may be evaluated in this article, or claim that may be made by its manufacturer, is not guaranteed or endorsed by the publisher.
